# Correction: Microbial Gene Ontology informed deep neural network for microbe functionality discovery in human diseases

**DOI:** 10.1371/journal.pone.0296316

**Published:** 2023-12-19

**Authors:** Yunjie Liu, Yao-zhong Zhang, Seiya Imoto

The last author, Seiya Imoto, should also be listed as an additional corresponding author. Seiya Imoto’s email address is imoto@hgc.jp.

In the acknowledgments section, the following statement should be included: The super-computing resource was provided by the Human Genome Center, Institute of Medical Science, The University of Tokyo (https://gc.hgc.jp/en/).

The following information is missing from the Funding statement: This research was supported by Grant-in-Aid for Scientific Research (Grant Number 21H03538 to S.I.) from Japan Society for the Promotion of Science and the Uehara Memorial Foundation (to S.I.). The computing resources were provided by Human Genome Center, the Institute of Medical Science, the University of Tokyo.
