# Supplementary material for: Microbial Gene Ontology informed deep neural network for microbe functionality discovery in human diseases
Source: PLoS One. 2023 Aug 21;18(8):e0290307. doi: 10.1371/journal.pone.0290307 (PMC10441785; doi:10.1371/journal.pone.0290307)
Supplement: Attachment — Submitted filename: Response to Reviewers.docx [file pone.0290307.s001.docx]

Responds to the reviewers’ comments

We sincerely thank all reviewers on the valuable comments on this manuscript.

Reviewer #1: This article presents an algorithm for inferring gene function based on gene abundance, which bears a striking resemblance to the P-Net algorithm published in Nature in 2021. While the algorithm is innovative, the lack of many methodological details in the article raises doubts about its value. Here are a few questions I am concerned about.

1. In the left side of Figure 1, the process for obtaining the abundance of each gene is shown. Therefore, the text "Metagenome sequence" should be corrected to "sequencing reads" instead of "genome"."

[Liu response]: Thank you for pointing this out. The text has been corrected in the Figure 1.

2. How does the author obtain the GO annotation for each UHGG gene? The author only mentions “The GO annotation information is obtained by referring to the UniProt database”. Using blastp? What parameters are used?

[Liu response]: Thank you for pointing this out. First, we obtain the protein annotation from each gene provided by the UHGG and find out the gene-protein mapping. Then, we search the Gene Ontology annotation of each protein from the Uniprot database to find the out the protein-GO mapping. Finally, by merging the gene-protein mapping and protein-GO mapping, we obtain the GO annotation for each UHGG gene.

3. The author claims “We prepare a gene set where the genes exist in more than 1% of 67 UHGG samples as commonly existing genes”. What is the detail? How many reads or TPM are regards as “exist in a sample”. As we know, some low abundance genes may be due to the sequencing error.

[Liu response]: Thank you for pointing this out. The number of genes selected in the gene set where the genes exist in more than 1% of UHGG samples is 22,927. Considering we have filtered the gene set by using commonly existing genes, genes of TPM>0 were regarded as existing in a sample.

4. Figure 1 shows the depth of the GO-informed neural network is 4 layers (exclude input layer). However, in line 95-97, the authors mention “The second layer to the seventh layer”. So, how many layers are used? The author should correct the error in the figure 1.

[Liu response]: Thank you for pointing this out. The complete GO-informed neural network includes 1 input layer, 6 GO hierarchical structure layers form GO level 6 to GO level 1, and 1 predictive layer. We have corrected the GO hierarchical structure layers part in the Figure 1.

5. Formula: y = f[(M ∗W)T xlayer_i +b] (i = 6, 5, 4, 3, 2, 1) shows how the connection between layers. M is the mask matrix is dependent on the GO annotation of the genes, which can’t be trained. The trainable parameters should be W and b. The author should add more sentences to illustrate the GO network.

[Liu response]: Thank you for your suggestion. We have added the description of GO network in the section "GO network informed DNN construction".

6. What is the y0? How did the author prepare the target?

[Liu response]: Thank you for pointing this out. The DeepLIFT-based important scores were calculated to visualize the important genes and GO terms in a specific disease. Therefore, we use the whole dataset in a specific disease as the target to calculate the important scores. In this case, y0 will be the disease label of whole dataset in a specific disease.

7. How did the network train? Which Loss function, optimization algorithm? The author should provide a figure to show the training process (Loss vs epoch).

[Liu response]: Thank you for pointing this out. The loss function is binary crossentropy function and the optimization algorithm is Adam optimizer. We have added the description in the "Parameters settings" section. The training curves for are shown in the Supplemental Figure 1. ~ Supplemental Figure 4.

Supplemental Figure 1. The training curves in T2D. A: training loss and validation loss vs epoch. B: F1-score vs epoch in training set in each layer. C: F1-score vs epoch in validation set in each layer.

Supplemental Figure 2. The training curves in LC. A: training loss and validation loss vs epoch. B: F1-score vs epoch in training set in each layer. C: F1-score vs epoch in validation set in each layer.

Supplemental Figure 3. The training curves in IBD. A: training loss and validation loss vs epoch. B: F1-score vs epoch in training set in each layer. C: F1-score vs epoch in validation set in each layer.

Supplemental Figure 4. The training curves in CRC. A: training loss and validation loss vs epoch. B: F1-score vs epoch in training set in each layer. C: F1-score vs epoch in validation set in each layer.

8. The author should make the source code to open access.

[Liu response]: Thank you for pointing this out. We have made the source code to open access.

Reviewer #2: I have reviewed your paper and found it to be interesting and informative. However, there are several issues that need to be addressed before the paper can be accepted for publication.

1. The arrangement of the figure caption and table caption is in a mess. Please rearrange them.

[Liu response]: Thank you for pointing this out. We have adjusted the figure caption and table caption.

2. Regarding your evaluation protocol, it would be helpful to know how you divided the training+validation and testing sets. Did you do this randomly or intentionally? If you swap the training+validation and testing, what would be the resulting performance?

[Liu response]: Thank you for pointing this out. The training+validation and testing sets are split randomly. Since the sample size is small and we use 9:1 split ratio to split training+validation and testing, if we swap the training+validation and testing, the samples to be learned will be very small and the model will underfitted in this case.

3. Why did you use a 9:1 split ratio? Additionally, besides the evaluation metrics provided, could you provide a biological interpretation to help illustrate the reliability of your model?

[Liu response]: Thank you for pointing this out. Considering the sample size is small, we use a 9:1 split ratio to make the model to learn well. In the "GO informed neural network visualization" section, we have provided the example biological interpretation in T2D and IBD diseases.

4. For the SVM kernel, did you try using the RBF kernel? If so, how did its performance compare to your method? Also, have you tried using XGBoost? As it is known to achieve state-of-the-art performance compared to other machine learning methods.

[Liu response]: Thank you for pointing this out. In SVM kernel, we test only linear and polynomial kernel. For XGBoost, RF and XGBoost are both decision tree algorithms and RF have been shown good performance in previous research about using metagenome sequence-based feature to do the disease prediction. Therefore, we only test the performance of RF in our study.

5. Could you please provide information on your beta and lambda grid search settings for your elastic net model?

[Liu response]: Thank you for pointing this out. We did not perform the grid search on beta and lambda for the elastic net penalty in the logistic regression model. We use the default settings of l1 ratio=0.5. Instead, we test l1 and l2 penalty as the special cases of the elastic net model.

6. AutoNN achieves worse performance than other methods. Have you tried combining GO with other machine learning or statistical learning methods that you benchmarked? It may not be necessary to use a deep learning model like AutoNN.

[Liu response]: Thank you for pointing this out. Our GO model did not combine with AutoNN or any other machine learning models. AutoNN was used as a representative NN model to compare the performance of our model and other machine learning models.

7. The precision of 0.522 to 0.698 are relatively low, indicating a high number of false positives in the classification of T2D. Could you explain why this may be the case?

[Liu response]: Thank you for pointing this out. In T2D dataset, it is a difficult dataset from the performance of different models (precision from 0.522 to 0.698). Combining with the training curve in Supplemental Figure. 1, we find the model overfitted in this dataset and it cannot generate well in the validation set. One main reason of this case is that T2D is a complex disease and the disease is not only related to the gut microbiome, but also related to other factors such as human genetics.

8. I recommend using DeepLift to calculate the feature importance of your model. I want to see the shap values comparison from the feature of your AutoNN. Also, please apply SHAP method to explain those machine learning and statistical learning methods that you benchmarked.

[Liu response]: Thank you for pointing this out. To compare the DeepLift importance score in our model and other model's SHAP value, we calculate the SHAP value of AutoNN, RF, and SVM in T2D and IBD dataset separately using SHAP (Lundberg, S. M., & Lee, S. I. (2017). A unified approach to interpreting model predictions. Advances in neural information processing systems, 30.). We plot top 10 SHAP value in different models (see Supplemental Figure. 5 and Supplemental Figure. 6).

Supplemental Figure 5. T2D top 10 SHAP value in different models. A: T2D top 10 SHAP value in AutoNN. B: T2D top 10 SHAP value in RF. C: T2D top 10 SHAP value in SVM.

Supplemental Figure 6. IBD top 10 SHAP value in different models. A: IBD top 10 SHAP value in AutoNN. B: IBD top 10 SHAP value in RF. C: IBD top 10 SHAP value in SVM.

From the SHAP value and DeepLift results (Figure 3. and Figure 4), we find that GO-NN DeepLift-important-score based important genes are different by comparing with other models. This is because our important genes are related to the GO network while other models do not have this constraint. Moreover, our model not only can find the important genes, but also can find the important GO terms and the relation between genes and GO terms, which can not be visualized in SHAP value in other models.

9. Could you please provide the data URL in your Github? I was unable to locate it, and why did you only provide T2D and LC data loader codes? Where are the IBD and CRC data?

[Liu response]: Thank you for pointing this out. We have updated the Github page and add the data URL. We also provide IBD and CRC data loader codes and the data.

10. Please included a comparison with other state-of-the-art methods that predict human disease from microbiota, such as those presented in "Multimodal deep learning applied to classify healthy and disease states of human microbiome" (<https://www.nature.com/articles/s41598-022-04773-3>) and "DeepMicro: deep representation learning for disease prediction based on microbiome data" (<https://www.nature.com/articles/s41598-020-63159-5>). It would provide a more comprehensive understanding of the performance of the proposed method in comparison to other relevant approaches.

[Liu response]: While we appreciate the suggestions, we respectfully disagree for the following reasons. First, in these models, the feature they prepared is different with ours. In "Multimodal deep learning applied to classify healthy and disease states of human microbiome" (<https://www.nature.com/articles/s41598-022-04773-3>), they use bacterial proportion, metabolic function abundance, and genome-level abundance; In "DeepMicro: deep representation learning for disease prediction based on microbiome data" (<https://www.nature.com/articles/s41598-020-63159-5>), they use strain-level marker profile and species-level relative abundance profile. Second, these models are not designed for microbe functionality discovery, which is a major objective of our model.
